# Seroepidemiology of *Helicobacter Pylori* Infection in Pregnant Women in Rural Durango, Mexico

**Published:** 2013-12

**Authors:** Cosme Alvarado-Esquivel

**Affiliations:** Biomedical Research Laboratory, Faculty of Medicine and Nutrition, Juárez University of Durango State, Avenida Universidad S/N. 34000 Durango, Dgo, Mexico

**Keywords:** Epidemiology, infection, cross-sectional study, risk factors, serology

## Abstract

The seroepidemiology of *Helicobacter pylori* infection in pregnant women in Durango, Mexico is largely unknown. The prevalence of anti-*H. pylori* IgG antibodies was examined in 343 pregnant women living in rural areas in 7 municipalities in Durango State, Mexico, using enzyme-linked immunosorbent assays (ELISA). A correlation of *H. pylori* seropositivity with socio-demographic, obstetric and behavioral characteristics of pregnant women was also assessed. In total, 179 (52.2%) of the 343 pregnant women (mean age, 24.2 ± 5.9 years) had *H. pylori* IgG antibodies, 75 (41.9%) of whom had *H. pylori* IgG antibody levels higher than 100 U/mL. The seroprevalence of *H. pylori* infection varied from 33.3% to 65% among municipalities. In contrast, the seroprevalence was comparable among women regardless their age, educational level, occupation, socioeconomic status, animal contacts, foreign travel, eating habits, contact with soil, crowding, sanitary conditions at home and educational level of the head of their families. Multivariant analysis of socio-demographic and behavioral variables showed that *H. pylori* seropositivity was associated with municipality (OR=1.12; 95% CI: 1.01–1.24; *P*=0.02). Of the obstetric characteristics, the seroprevalence of* H. pylori* infection increased significantly with the number of pregnancies and deliveries but not with the number of cesarean sections or miscarriages. Rural pregnant women in Durango had a lower seroprevalence of *H. pylori* infection than those from populations in developing countries. Results support a variability of *H. pylori* seroprevalence within a region. Further research at a municipal level might help to understand the epidemiology of *H. pylori* infection.

## INTRODUCTION

The bacterium *Helicobacter pylori* causes infections in humans all around the world (1). About one-half of the world’s population has been exposed to *H. pylori* (1, 2). It remains unclear how *H. pylori* is transmitted to humans. However, it is likely that *H. pylori* can be transmitted by the following routes: person-to-person (3), oral-oral or fecal-oral (4), and consumption of contaminated water (4, 5). Vertical transmission of *H. pylori* through breast-feeding may also occur (6). Most individuals infected with *H. pylori* remain asymptomatic (7). However, infections with *H. pylori* may lead to gastric (1, 2, 8, 9) and extra gastric (10, 11) diseases. The seroprevalence of *H. pylori* infection varies substantially among countries. For instance, seroprevalences from 15.1% to 32.5% have been reported in Australia (12), Saudi Arabia (13), and the USA (14), while seroprevalences from 43% to 66.4% have been reported in Korea (15), Israel (16), Germany (17), Italy (18), Greenland (19), and Iran (20). The level of country development influences the seroprevalence. The seroprevalence is higher in developing than in developed countries (21).

The seroepidemiology of *H. pylori* infection in Mexico in general and in the northern Mexican state of Durango in particular has been poorly explored. There is a lack of information about the seroprevalence of anti-*H. pylori* antibodies in pregnant women in rural Mexico. Many pregnant women in rural areas in Mexico live under suboptimal housing and sanitary conditions including crowding and poor availability of potable water and sewage disposal that may favor transmission of *H. pylori*. Therefore, this survey was aimed to determine the seroprevalence of *H. pylori* infection in pregnant women in rural areas in Durango, Mexico, and to determine socio-demographic, obstetric, and behavioral characteristics of the pregnant women associated with *H. pylori* seropositivity.

## METHODS

### Selection and description of participants

Through a cross-sectional study using serum samples of a previous *Toxoplasma gondii* survey (22), 343 pregnant women living in rural areas in Durango, Mexico were studied. Inclusion criteria for the pregnant women were: 1) living in rural Durango and 2) aged 13 years and older. Exclusion criterion was women with any missing data. Pregnant women studied had from 1 to 9 months of pregnancy, and their mean age was 24.2 ± 5.9 years (in a range 13–42 years). Sera were collected from August 2007 to February 2008.

### Technical information

Socio-demographic data including age, birth place, municipality of residence, occupation, educational level, socio-economic status and housing conditions were obtained from all participants. Housing conditions were obtained by using the Bronfman’s criteria (23) and allowed to assess crowding and sanitation. Briefly, five variables were evaluated: number of persons in the house, number of rooms in the house, material of the floor of the house, availability of drinkable water, and form of elimination of excretes. In addition, educational level of the head of the family was obtained. Obstetric history (pregnancies, deliveries, caesarean sections, and miscarriages) was also obtained from each woman. Behavioural data including animal contacts, foreign travel, frequency of meat consumption, type of meat consumption (pork, lamb, beef, goat, boar, chicken, turkey, rabbit, deer, squirrel, horse, snake and fish), degree of meat cooking, consumption of unpasteurized milk, untreated water, unwashed raw vegetables or fruits, contact with soil (gardening or agriculture), and frequency of eating away from home (restaurants or fast food outlets) from all pregnant women studied were obtained.

Serum samples of pregnant women were examined for detection of anti-*H. pylori* IgG antibodies using a commercially available enzyme-linked immunosorbent assay (ELISA) kit, Anti-*H. pylori* IgG AccuBind ELISA (Monobind Inc, Lake Forest, California). Anti-*H. pylori* IgG antibody levels were expressed as Units (U)/mL, and a value higher than 20 U/mL was considered a positive result. An ELISA U/mL was a unit for measuring concentration of anti-*H. pylori* IgG antibodies as defined in the reference standards for the ELISA method used. By utilizing serum references of known antibody activity at 0, 10, 25, 50 and 100 U/mL of IgG, a reference curve was generated from which the antibody concentration of the samples was ascertained. Assay results were considered valid when the maximum absorbance (100 U/mL calibrator) was higher than 1.3. The ELISA was performed following the instructions of the manufacturer.

This study was approved by the Ethical Committee of the Instituto de Seguridad y Servicios Sociales de los Trabajadores del Estado in Durango City. Mexico.

### Statistics

Statistical analysis was performed with the Epi Info version 3.5.4 software and SPSS version 15.0 software. For calculation of the sample size, a reference seroprevalence of 50.7% (24) as the expected frequency for the factor under study, 25000 as the population size from which the sample was selected, 56% as the least acceptable result, and a 95% confidence level were considered. The result of the sample size calculation was 337 women. Frequencies between groups were compared with the Pearson’s chi-square test and the Fisher exact test (when values were less than 5). Bivariate and multivariate analyses were used to assess the association between *H. pylori* seropositivity and women characteristics. Variables were included in the multivariate analysis if they had a *P* value ≤0.20 in the bivariate analysis. Odd ratio (OR) and 95% confidence interval (CI) were calculated by multivariate analysis, using the Enter method. Results were considered statistically significant at a *P* value <0.05.

## RESULTS

**Figure 1:**
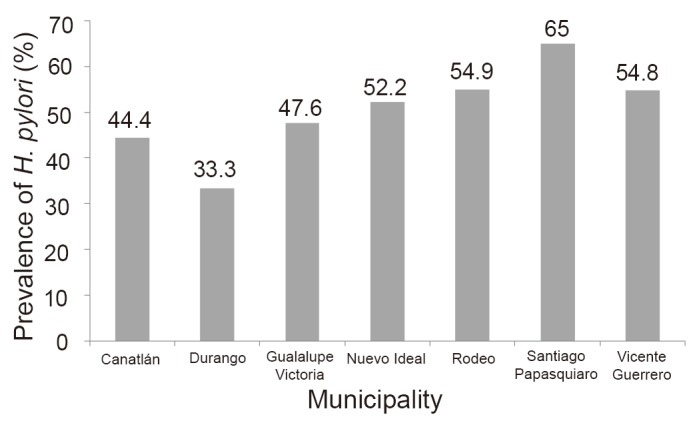
Seroprevalence of *H. pylori* infection in pregnant women in the seven municipalities studied. The seroprevalence varied significantly (*P*=0.02) among municipalities.

*H. pylori* IgG antibodies were detected in 179 (52.2%) of the 343 pregnant women studied. Of the 179 *H. pylori* IgG positive women, 75 (41.9%) had IgG levels higher than 100 U/mL, 32 (17.9%) between 51 to 100 U/mL, and 72 (40.2%) between 21 to 50 U/mL. The mean seroprevalence of *H. pylori* infection among municipalities was 50.3% (SD=9.9); and seroprevalence varied from 33.3% in women in the Durango municipality to 65% in women in the Santiago Papasquiaro municipality (Fig. 1). In contrast, seroprevalence was comparable among women regardless their age, educational level, occupation, socioeconomic status, and educational level of the head of the family. Of the behavioral characteristics, women who consumed beef had a significantly higher seroprevalence of *H. pylori* infection than those who did not consume such meat (53.5% versus 27.7%, respectively). Other behavioral characteristics including animal contacts, foreign travel, frequency of meat consumption, consumption of meat other than beef, degree of meat cooking, consumption of unpasteurized milk, untreated water, unwashed raw vegetables or fruits, contact with soil, and frequency of eating away from home were not found associated with *H. pylori* seropositivity. None of the housing conditions including number of persons in the house, number of rooms in the house, material of the floor of the house, availability of drinkable water, and form of elimination of excretes were found associated with *H. pylori* seropositivity.

Multivariate analysis of socio-demographic and behavioral variables with a *P* value ≤0.20 including age (*P*=0.11), municipality (*P*=0.005), educational level (*P*=0.12), occupation (*P*=0.19), consumption of beef (*P*=0.03), goat (*P*=0.16), unpasteurized cow milk (*P*=0.18), unpasteurized goat milk (*P*=0.13), and availability of drinkable water (*P*=0.17), showed that *H. pylori* seropositivity was only positively associated with municipality (OR=1.12; 95% CI: 1.01–1.24; *P*=0.02) (Table 1).

**Table 1:** Results of the multivariate regression analysis

Variable	*P* value	Odds ratio	95% Confidence interval

Age	0.1	1.03	0.99-1.07
Municipality	0.02	1.12	1.01-1.24
Education	0.27	0.63	0.28-1.42
Occupation	0.19	1.59	0.78-3.21
Consumption			
Beef	0.1	2.46	0.83-7.22
Goat meat	0.24	1.69	0.70-4.08
Raw cow milk	0.13	0.71	0.45-1.11
Raw goat milk	0.16	0.36	0.08-1.51
Availability of drinkable water	0.66	1.1	0.70-1.72

**Figure 2:**
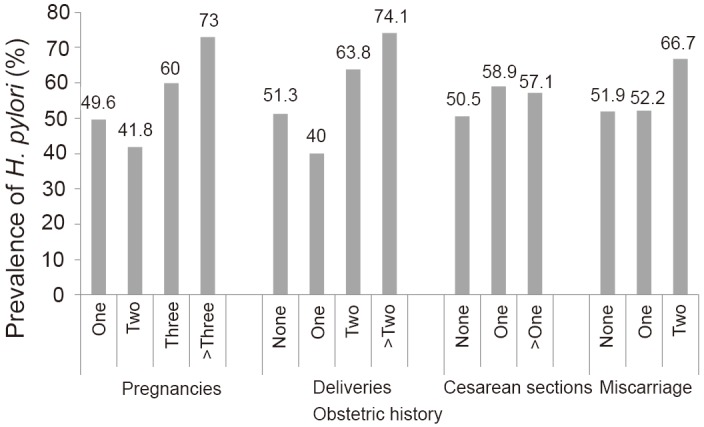
Correlation of obstetric history and seroprevalence of *H. pylori* infection in the pregnant women studied. Seroprevalence varied significantly with the number of pregnancies (*P*=0.005) and deliveries (*P*=0.005).

Of the obstetric characteristics, the seroprevalence of *H. pylori* infection increased significantly with the number of pregnancies (*P*=0.005) and deliveries (*P*=0.005) (Fig. 2). In contrast, the seroprevalence was comparable among women regardless their histories of cesarean sections and miscarriages.

## DISCUSSION

The 52.2% seroprevalence of *H. pylori* infection in the pregnant women of rural Durango found in the present study is lower than the mean national seroprevalence (66%) reported in general population in Mexico (25). However, interpretation of such comparison should be taken with care since differences in laboratory tests and characteristics of the studied populations between the studies exist. A commercial ELISA kit was used in the present study, and a homemade ELISA kit was used for the national survey. In the present study, only rural pregnant women were surveyed while men and women of urban and rural communities were studied in the national survey. The seroprevalence of *H. pylori* infection has been found lower in subjects living in rural areas than those living in urban areas (26). Therefore, this fact might explain the lower seroprevalence found in rural pregnant women than the 66% mean national seroprevalence reported in Mexico (25). To the best of my knowledge, this study is the first one conducted in pregnant women in rural Mexico. In a regional context, the seroprevalence of *H. pylori* infection found in rural pregnant women is comparable with a 50.7% seroprevalence found in a Mennonite community (24), but lower than a 66% seroprevalence found in Tepehuanos in Durango (27). These three regional surveys studied rural communities and used the same commercial ELISA kit. The difference in the seroprevalence between pregnant women and Tepehuanos might be due to differences in the general characteristics of the subjects studied. The current study included only pregnant women while the study in Tepehuanos included both men and women. There is only one previous study about the seroepidemiology of *H. pylori* in Mexican pregnant women. Goodman *et al* (28) studied pregnant women from a USA-Mexico border population and found a 74% seroprevalence in women from the Mexican side and a 56% seroprevalence in women from the USA side. In an international context, the seroprevalence of *H. pylori* infection in rural pregnant women in Durango is lower than the estimated 80% to 90% seroprevalence of *H. pylori* infection in developing countries (21). In addition, the seroprevalence of *H. pylori* infection found in the current study is higher than that (21.5%) reported in pregnant women in France (29), and comparable to the 45.9% reported in pregnant women in Israel (30). A number of direct and indirect methods to detect *H. pylori* infection exist. Direct methods to detect *H. pylori* including ELISA for *H. pylori* antigen and polymerase chain reaction for *H. pylori* DNA are used to detect active infections. However, PCR cannot distinguish between living or dead organisms (31). On the other hand, ELISA for detecting anti-*H pylori* antibodies is an indirect method widely used for determination of *H. pylori* exposure. The use of the latter indirect method for diagnosis of *H. pylori* infection is a good strategy for detecting both current and past infections. Direct detection of *H. pylori* by culture, polymerase chain reaction and sequencing from biopsy material has been performed in Mexico, and genotyping of *H. pylori* isolates showed that all strains were vacA+ and clustered in eight genetic groups depending of the presence of iceA1 and iceA2 or both genes (32). In addition, “triple-positive” (vacA, cagA, babA genes) strains of *H. pylori* have been found in Mexico (32) and Cuba (33).

Concerning socio-demographic and behavioral characteristics in rural pregnant women, multivariate analysis showed that *H. pylori* seropositivity was only associated with the variable municipality. It is not clear why seroprevalence of *H. pylori* varied among municipalities. It is likely that differences in sanitation among the municipalities might explain the differences in the seroprevalences. However, further analysis by collapsing the housing conditions into subgroups yielded a limited number of participants for comparison and did not allow drawing clear conclusions on differences among municipalities. To the best of my knowledge, no previous serosurvey of *H. pylori* infection had explored the seroprevalence at a municipality level. Other putative factors associated with *H. pylori* infection including age (3, 16, 34), low educational level (35), socioeconomic status (3, 25), laborer occupation (27), and crowding (35) were not found associated with *H. pylori* seropositivity in the present study.

Concerning obstetric data, the seroprevalence of* H. pylori* increased significantly with the number of pregnancies and deliveries. This finding is consistent with previous reports (24, 30, 36). In contrast, seropositivity to *H. pylori* was not associated with cesarean sections or abortions. In a previous study in the region, abortion history was associated with *H. pylori* seropositivity in female Mennonites (24). It is not clear why such association occurred in Mennonites but not in rural pregnant women. On the other hand, the lack of such association in the present study is consistent with previous findings in female Tepehuanos in Durango (27). Further research to determine the association of *H. pylori* infection and obstetric history in women is needed.

## CONCLUSIONS

Rural pregnant women in Durango have a lower seroprevalence of *H. pylori* infection than the one estimated for populations in developing countries. Rural pregnant women in Durango have a similar or lower seroprevalence of *H. pylori* infection than those found in other rural communities in the region. Results support a variability of *H. pylori* seroprevalence within a region (OR=1.12; 95% CI: 1.01–1.24; *P*=0.02). Further research at a municipal level might help to understand the epidemiology of *H. pylori* infection.
